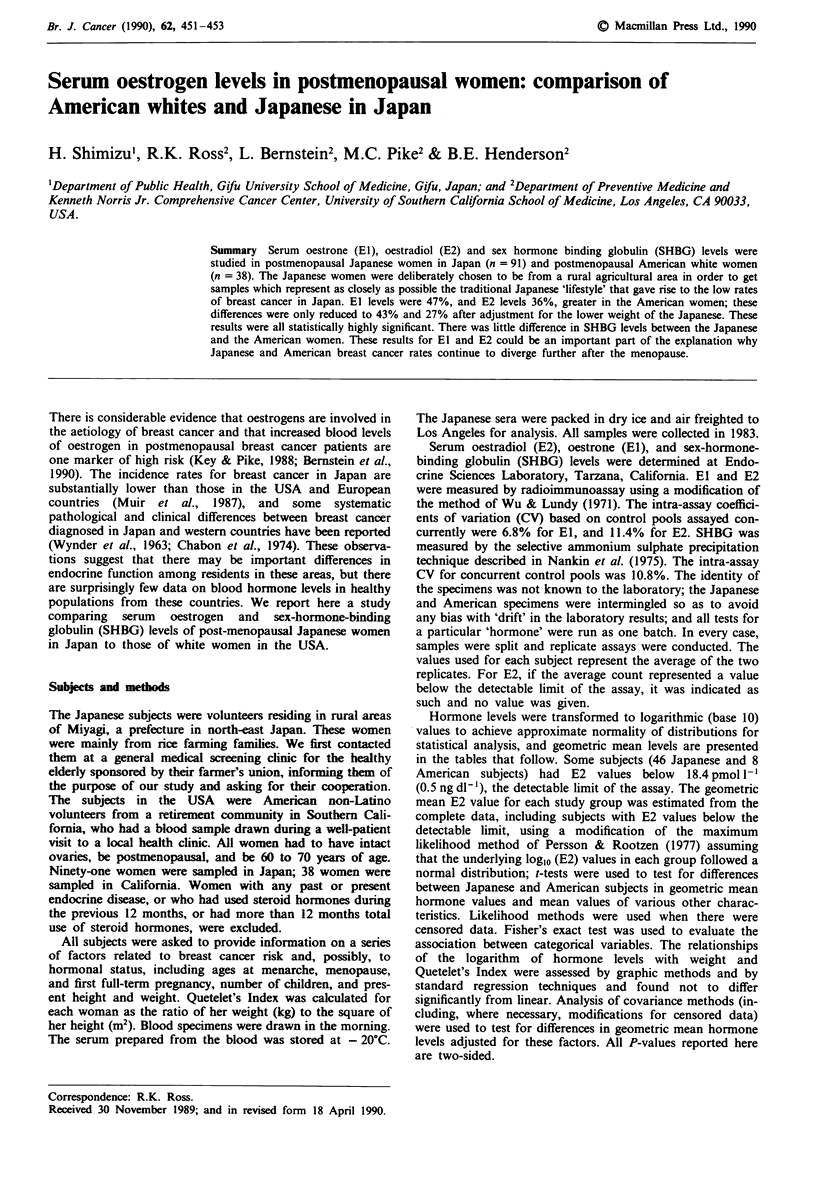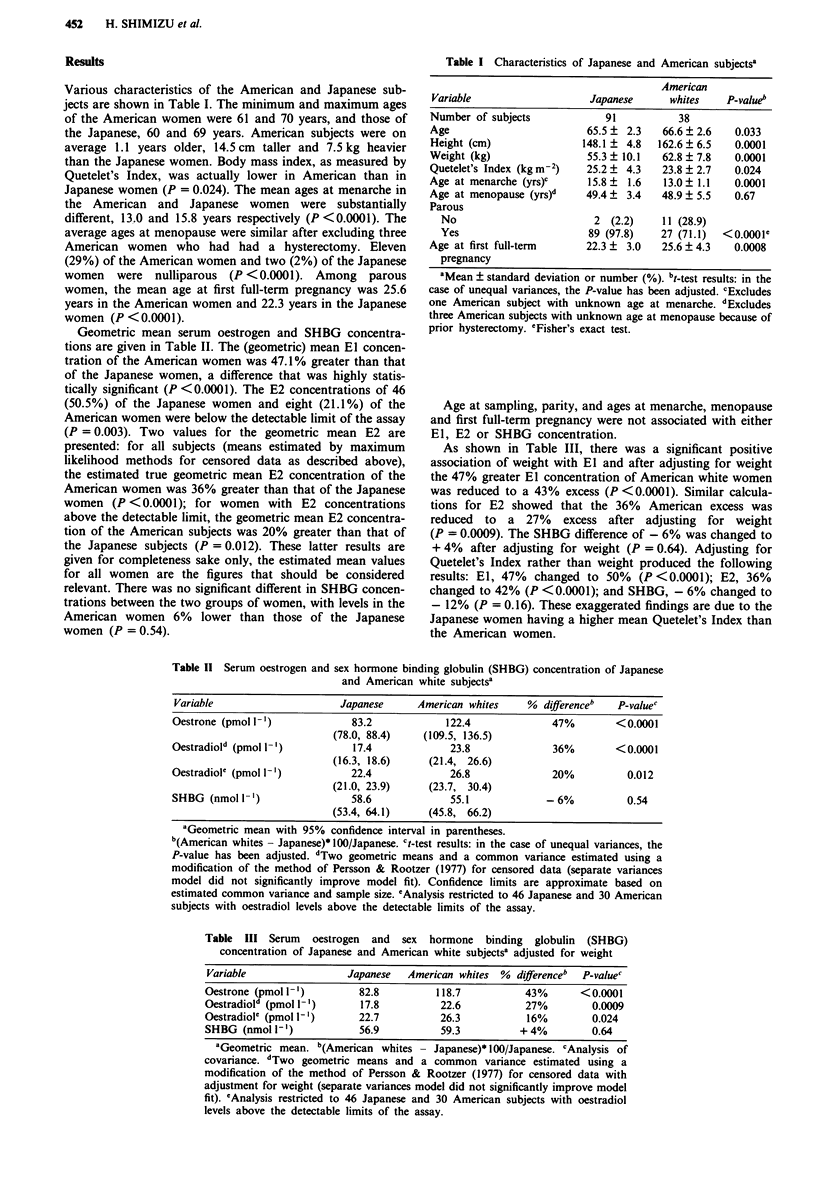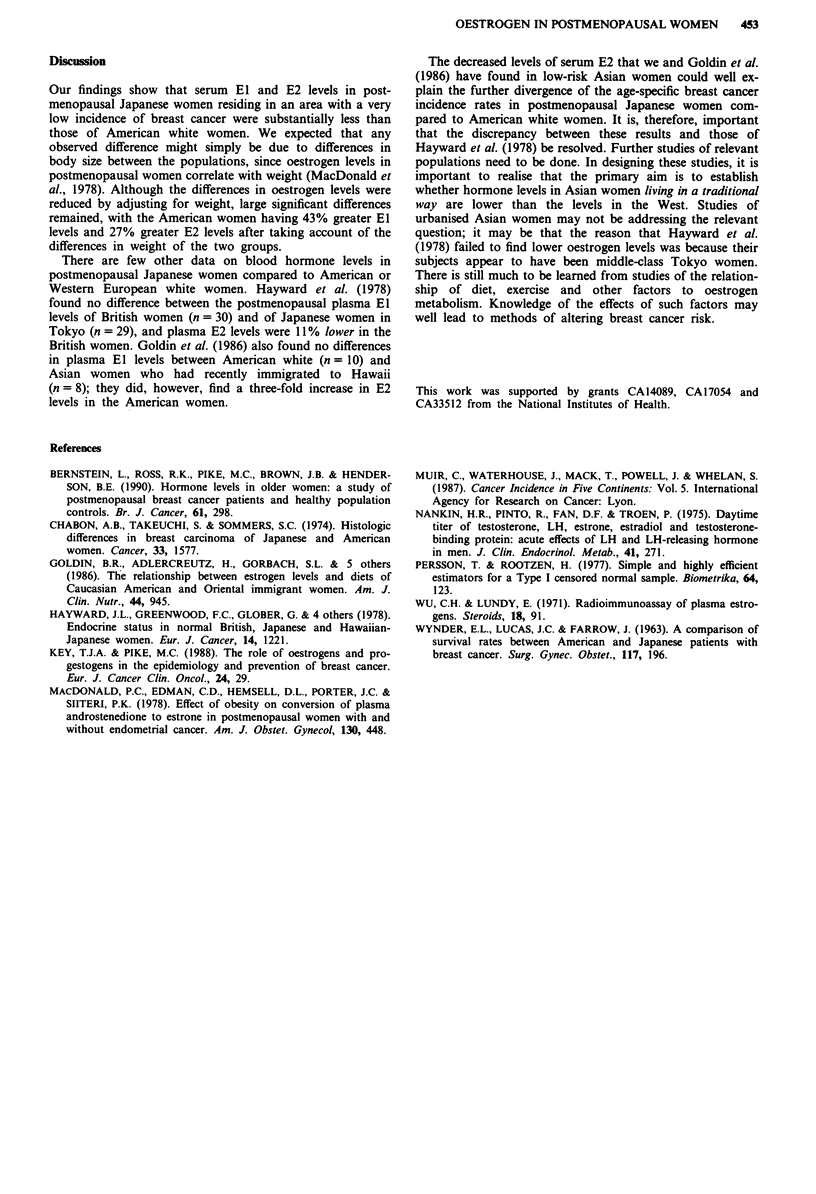# Serum oestrogen levels in postmenopausal women: comparison of American whites and Japanese in Japan.

**DOI:** 10.1038/bjc.1990.316

**Published:** 1990-09

**Authors:** H. Shimizu, R. K. Ross, L. Bernstein, M. C. Pike, B. E. Henderson

**Affiliations:** Department of Public Health, Gifu University School of Medicine, Japan.

## Abstract

Serum oestrone (E1), oestradiol (E2) and sex hormone binding globulin (SHBG) levels were studied in postmenopausal Japanese women in Japan (n = 91) and postmenopausal American white women (n = 38). The Japanese women were deliberately chosen to be from a rural agricultural area in order to get samples which represent as closely as possible the traditional Japanese 'lifestyle' that gave rise to the low rates of breast cancer in Japan. E1 levels were 47%, and E2 levels 36%, greater in the American women; these differences were only reduced to 43% and 27% after adjustment for the lower weight of the Japanese. These results were all statistically highly significant. There was little difference in SHBG levels between the Japanese and the American women. These results for E1 and E2 could be an important part of the explanation why Japanese and American breast cancer rates continue to diverge further after the menopause.


Br. J. Cancer (1990), 62, 451-453 © Macmillan Press Ltd., 1990

### Serum oestrogen levels in postmenopausal women: comparison of American whites and Japanese in Japan

H. Shimizu[1], R.K. Ross[2], L. Bernstein[2], M.C. Pike[2] & B.E. Henderson[2]

[1]*Department of Public Health, Gifu University School of Medicine, Gifu, Japan; and* [2]*Department of Preventive Medicine and Kenneth Norris Jr. Comprehensive Cancer Center, University of Southern California School of Medicine, Los Angeles, CA 90033, USA.*

**Summary** Serum oestrone (E1), oestradiol (E2) and sex hormone binding globulin (SHBG) levels were studied in postmenopausal Japanese women in Japan ($n = 91$) and postmenopausal American white women ($n = 38$). The Japanese women were deliberately chosen to be from a rural agricultural area in order to get samples which represent as closely as possible the traditional Japanese 'lifestyle' that gave rise to the low rates of breast cancer in Japan. E1 levels were 47%, and E2 levels 36%, greater in the American women; these differences were only reduced to 43% and 27% after adjustment for the lower weight of the Japanese. These results were all statistically highly significant. There was little difference in SHBG levels between the Japanese and the American women. These results for E1 and E2 could be an important part of the explanation why Japanese and American breast cancer rates continue to diverge further after the menopause.

There is considerable evidence that oestrogens are involved in the aetiology of breast cancer and that increased blood levels of oestrogen in postmenopausal breast cancer patients are one marker of high risk (Key & Pike, 1988; Bernstein *et al.*, 1990). The incidence rates for breast cancer in Japan are substantially lower than those in the USA and European countries (Muir *et al.*, 1987), and some systematic pathological and clinical differences between breast cancer diagnosed in Japan and western countries have been reported (Wynder *et al.*, 1963; Chabon *et al.*, 1974). These observations suggest that there may be important differences in endocrine function among residents in these areas, but there are surprisingly few data on blood hormone levels in healthy populations from these countries. We report here a study comparing serum oestrogen and sex-hormone-binding globulin (SHBG) levels of post-menopausal Japanese women in Japan to those of white women in the USA.

### Subjects and methods

The Japanese subjects were volunteers residing in rural areas of Miyagi, a prefecture in north-east Japan. These women were mainly from rice farming families. We first contacted them at a general medical screening clinic for the healthy elderly sponsored by their farmer's union, informing them of the purpose of our study and asking for their cooperation. The subjects in the USA were American non-Latino volunteers from a retirement community in Southern California, who had a blood sample drawn during a well-patient visit to a local health clinic. All women had to have intact ovaries, be postmenopausal, and be 60 to 70 years of age. Ninety-one women were sampled in Japan; 38 women were sampled in California. Women with any past or present endocrine disease, or who had used steroid hormones during the previous 12 months, or had more than 12 months total use of steroid hormones, were excluded.

All subjects were asked to provide information on a series of factors related to breast cancer risk and, possibly, to hormonal status, including ages at menarche, menopause, and first full-term pregnancy, number of children, and present height and weight. Quetelet's Index was calculated for each woman as the ratio of her weight (kg) to the square of her height ($m^2$). Blood specimens were drawn in the morning. The serum prepared from the blood was stored at $-20°C$. The Japanese sera were packed in dry ice and air freighted to Los Angeles for analysis. All samples were collected in 1983.

Serum oestradiol (E2), oestrone (E1), and sex-hormone-binding globulin (SHBG) levels were determined at Endocrine Sciences Laboratory, Tarzana, California. E1 and E2 were measured by radioimmunoassay using a modification of the method of Wu & Lundy (1971). The intra-assay coefficients of variation (CV) based on control pools assayed concurrently were 6.8% for E1, and 11.4% for E2. SHBG was measured by the selective ammonium sulphate precipitation technique described in Nankin *et al.* (1975). The intra-assay CV for concurrent control pools was 10.8%. The identity of the specimens was not known to the laboratory; the Japanese and American specimens were intermingled so as to avoid any bias with 'drift' in the laboratory results; and all tests for a particular 'hormone' were run as one batch. In every case, samples were split and replicate assays were conducted. The values used for each subject represent the average of the two replicates. For E2, if the average count represented a value below the detectable limit of the assay, it was indicated as such and no value was given.

Hormone levels were transformed to logarithmic (base 10) values to achieve approximate normality of distributions for statistical analysis, and geometric mean levels are presented in the tables that follow. Some subjects (46 Japanese and 8 American subjects) had E2 values below $18.4\,pmol\,l^{-1}$ ($0.5\,ng\,dl^{-1}$), the detectable limit of the assay. The geometric mean E2 value for each study group was estimated from the complete data, including subjects with E2 values below the detectable limit, using a modification of the maximum likelihood method of Persson & Rootzen (1977) assuming that the underlying $\log_{10}$ (E2) values in each group followed a normal distribution; $t$-tests were used to test for differences between Japanese and American subjects in geometric mean hormone values and mean values of various other characteristics. Likelihood methods were used when there were censored data. Fisher's exact test was used to evaluate the association between categorical variables. The relationships of the logarithm of hormone levels with weight and Quetelet's Index were assessed by graphic methods and by standard regression techniques and found not to differ significantly from linear. Analysis of covariance methods (including, where necessary, modifications for censored data) were used to test for differences in geometric mean hormone levels adjusted for these factors. All $P$-values reported here are two-sided.

Correspondence: R.K. Ross.
Received 30 November 1989; and in revised form 18 April 1990.

### Results

Various characteristics of the American and Japanese subjects are shown in Table I. The minimum and maximum ages of the American women were 61 and 70 years, and those of the Japanese, 60 and 69 years. American subjects were on average 1.1 years older, 14.5 cm taller and 7.5 kg heavier than the Japanese women. Body mass index, as measured by Quetelet's Index, was actually lower in American than in Japanese women ($P = 0.024$). The mean ages at menarche in the American and Japanese women were substantially different, 13.0 and 15.8 years respectively ($P < 0.0001$). The average ages at menopause were similar after excluding three American women who had had a hysterectomy. Eleven (29%) of the American women and two (2%) of the Japanese women were nulliparous ($P < 0.0001$). Among parous women, the mean age at first full-term pregnancy was 25.6 years in the American women and 22.3 years in the Japanese women ($P < 0.0001$).

Geometric mean serum oestrogen and SHBG concentrations are given in Table II. The (geometric) mean E1 concentration of the American women was 47.1% greater than that of the Japanese women, a difference that was highly statistically significant ($P < 0.0001$). The E2 concentrations of 46 (50.5%) of the Japanese women and eight (21.1%) of the American women were below the detectable limit of the assay ($P = 0.003$). Two values for the geometric mean E2 are presented: for all subjects (means estimated by maximum likelihood methods for censored data as described above), the estimated true geometric mean E2 concentration of the American women was 36% greater than that of the Japanese women ($P < 0.0001$); for women with E2 concentrations above the detectable limit, the geometric mean E2 concentration of the American subjects was 20% greater than that of the Japanese subjects ($P = 0.012$). These latter results are given for completeness sake only, the estimated mean values for all women are the figures that should be considered relevant. There was no significant different in SHBG concentrations between the two groups of women, with levels in the American women 6% lower than those of the Japanese women ($P = 0.54$).

**Table I** Characteristics of Japanese and American subjects[a]

| *Variable* | *Japanese* | *American whites* | *P-value*[b] |
|---|---|---|---|
| Number of subjects | 91 | 38 | |
| Age | 65.5 ± 2.3 | 66.6 ± 2.6 | 0.033 |
| Height (cm) | 148.1 ± 4.8 | 162.6 ± 6.5 | 0.0001 |
| Weight (kg) | 55.3 ± 10.1 | 62.8 ± 7.8 | 0.0001 |
| Quetelet's Index (kg m$^{-2}$) | 25.2 ± 4.3 | 23.8 ± 2.7 | 0.024 |
| Age at menarche (yrs)[c] | 15.8 ± 1.6 | 13.0 ± 1.1 | 0.0001 |
| Age at menopause (yrs)[d] | 49.4 ± 3.4 | 48.9 ± 5.5 | 0.67 |
| Parous | | | |
| No | 2 (2.2) | 11 (28.9) | |
| Yes | 89 (97.8) | 27 (71.1) | <0.0001[e] |
| Age at first full-term pregnancy | 22.3 ± 3.0 | 25.6 ± 4.3 | 0.0008 |

[a]Mean ± standard deviation or number (%). [b]*t*-test results: in the case of unequal variances, the *P*-value has been adjusted. [c]Excludes one American subject with unknown age at menarche. [d]Excludes three American subjects with unknown age at menopause because of prior hysterectomy. [e]Fisher's exact test.

Age at sampling, parity, and ages at menarche, menopause and first full-term pregnancy were not associated with either E1, E2 or SHBG concentration.

As shown in Table III, there was a significant positive association of weight with E1 and after adjusting for weight the 47% greater E1 concentration of American white women was reduced to a 43% excess ($P < 0.0001$). Similar calculations for E2 showed that the 36% American excess was reduced to a 27% excess after adjusting for weight ($P = 0.0009$). The SHBG difference of − 6% was changed to + 4% after adjusting for weight ($P = 0.64$). Adjusting for Quetelet's Index rather than weight produced the following results: E1, 47% changed to 50% ($P < 0.0001$); E2, 36% changed to 42% ($P < 0.0001$); and SHBG, − 6% changed to − 12% ($P = 0.16$). These exaggerated findings are due to the Japanese women having a higher mean Quetelet's Index than the American women.

**Table II** Serum oestrogen and sex hormone binding globulin (SHBG) concentration of Japanese and American white subjects[a]

| *Variable* | *Japanese* | *American whites* | *% difference*[b] | *P-value*[c] |
|---|---|---|---|---|
| Oestrone (pmol l$^{-1}$) | 83.2 (78.0, 88.4) | 122.4 (109.5, 136.5) | 47% | <0.0001 |
| Oestradiol[d] (pmol l$^{-1}$) | 17.4 (16.3, 18.6) | 23.8 (21.4, 26.6) | 36% | <0.0001 |
| Oestradiol[e] (pmol l$^{-1}$) | 22.4 (21.0, 23.9) | 26.8 (23.7, 30.4) | 20% | 0.012 |
| SHBG (nmol l$^{-1}$) | 58.6 (53.4, 64.1) | 55.1 (45.8, 66.2) | − 6% | 0.54 |

[a]Geometric mean with 95% confidence interval in parentheses.
[b](American whites − Japanese)*100/Japanese. [c]*t*-test results: in the case of unequal variances, the *P*-value has been adjusted. [d]Two geometric means and a common variance estimated using a modification of the method of Persson & Rootzer (1977) for censored data (separate variances model did not significantly improve model fit). Confidence limits are approximate based on estimated common variance and sample size. [e]Analysis restricted to 46 Japanese and 30 American subjects with oestradiol levels above the detectable limits of the assay.

**Table III** Serum oestrogen and sex hormone binding globulin (SHBG) concentration of Japanese and American white subjects[a] adjusted for weight

| *Variable* | *Japanese* | *American whites* | *% difference*[b] | *P-value*[c] |
|---|---|---|---|---|
| Oestrone (pmol l$^{-1}$) | 82.8 | 118.7 | 43% | <0.0001 |
| Oestradiol[d] (pmol l$^{-1}$) | 17.8 | 22.6 | 27% | 0.0009 |
| Oestradiol[e] (pmol l$^{-1}$) | 22.7 | 26.3 | 16% | 0.024 |
| SHBG (nmol l$^{-1}$) | 56.9 | 59.3 | + 4% | 0.64 |

[a]Geometric mean. [b](American whites − Japanese)*100/Japanese. [c]Analysis of covariance. [d]Two geometric means and a common variance estimated using a modification of the method of Persson & Rootzer (1977) for censored data with adjustment for weight (separate variances model did not significantly improve model fit). [e]Analysis restricted to 46 Japanese and 30 American subjects with oestradiol levels above the detectable limits of the assay.

### Discussion

Our findings show that serum E1 and E2 levels in postmenopausal Japanese women residing in an area with a very low incidence of breast cancer were substantially less than those of American white women. We expected that any observed difference might simply be due to differences in body size between the populations, since oestrogen levels in postmenopausal women correlate with weight (MacDonald *et al*., 1978). Although the differences in oestrogen levels were reduced by adjusting for weight, large significant differences remained, with the American women having 43% greater E1 levels and 27% greater E2 levels after taking account of the differences in weight of the two groups.

There are few other data on blood hormone levels in postmenopausal Japanese women compared to American or Western European white women. Hayward *et al.* (1978) found no difference between the postmenopausal plasma E1 levels of British women ($n = 30$) and of Japanese women in Tokyo ($n = 29$), and plasma E2 levels were 11% *lower* in the British women. Goldin *et al.* (1986) also found no differences in plasma E1 levels between American white ($n = 10$) and Asian women who had recently immigrated to Hawaii ($n = 8$); they did, however, find a three-fold increase in E2 levels in the American women.

The decreased levels of serum E2 that we and Goldin *et al.* (1986) have found in low-risk Asian women could well explain the further divergence of the age-specific breast cancer incidence rates in postmenopausal Japanese women compared to American white women. It is, therefore, important that the discrepancy between these results and those of Hayward *et al.* (1978) be resolved. Further studies of relevant populations need to be done. In designing these studies, it is important to realise that the primary aim is to establish whether hormone levels in Asian women *living in a traditional way* are lower than the levels in the West. Studies of urbanised Asian women may not be addressing the relevant question; it may be that the reason that Hayward *et al.* (1978) failed to find lower oestrogen levels was because their subjects appear to have been middle-class Tokyo women. There is still much to be learned from studies of the relationship of diet, exercise and other factors to oestrogen metabolism. Knowledge of the effects of such factors may well lead to methods of altering breast cancer risk.

This work was supported by grants CA14089, CA17054 and CA33512 from the National Institutes of Health.

### References

BERNSTEIN, L., ROSS, R.K., PIKE, M.C., BROWN, J.B. & HENDERSON, B.E. (1990). Hormone levels in older women: a study of postmenopausal breast cancer patients and healthy population controls. *Br. J. Cancer*, **61**, 298.

CHABON, A.B., TAKEUCHI, S. & SOMMERS, S.C. (1974). Histologic differences in breast carcinoma of Japanese and American women. *Cancer*, **33**, 1577.

GOLDIN, B.R., ADLERCREUTZ, H., GORBACH, S.L. & 5 others (1986). The relationship between estrogen levels and diets of Caucasian American and Oriental immigrant women. *Am. J. Clin. Nutr.*, **44**, 945.

HAYWARD, J.L., GREENWOOD, F.C., GLOBER, G. & 4 others (1978). Endocrine status in normal British, Japanese and Hawaiian-Japanese women. *Eur. J. Cancer*, **14**, 1221.

KEY, T.J.A. & PIKE, M.C. (1988). The role of oestrogens and progestogens in the epidemiology and prevention of breast cancer. *Eur. J. Cancer Clin. Oncol.*, **24**, 29.

MACDONALD, P.C., EDMAN, C.D., HEMSELL, D.L., PORTER, J.C. & SIITERI, P.K. (1978). Effect of obesity on conversion of plasma androstenedione to estrone in postmenopausal women with and without endometrial cancer. *Am. J. Obstet. Gynecol*, **130**, 448.

MUIR, C., WATERHOUSE, J., MACK, T., POWELL, J. & WHELAN, S. (1987). *Cancer Incidence in Five Continents:* Vol. 5. International Agency for Research on Cancer: Lyon.

NANKIN, H.R., PINTO, R., FAN, D.F. & TROEN, P. (1975). Daytime titer of testosterone, LH, estrone, estradiol and testosterone-binding protein: acute effects of LH and LH-releasing hormone in men. *J. Clin. Endocrinol. Metab.*, **41**, 271.

PERSSON, T. & ROOTZEN, H. (1977). Simple and highly efficient estimators for a Type I censored normal sample. *Biometrika*, **64**, 123.

WU, C.H. & LUNDY, E. (1971). Radioimmunoassay of plasma estrogens. *Steroids*, **18**, 91.

WYNDER, E.L., LUCAS, J.C. & FARROW, J. (1963). A comparison of survival rates between American and Japanese patients with breast cancer. *Surg. Gynec. Obstet.*, **117**, 196.